# Isolated Thyroid Metastasis from High-Grade Clear Cell Renal Cell Carcinoma After Radical Nephrectomy: A Case Report

**DOI:** 10.3390/reports9040346

**Published:** 2026-10-12

**Authors:** Constantinos Papadopoulos, Konstantina Dinaki, Rafail Ioannidis, Ioanna Gravalidou, Anna Zisi

**Affiliations:** 1 Department of ENT Surgery, General Hospital of Drama, 66132 Drama, Greece; constantine.mbg@gmail.com; 2 Medical School, Aristotle University of Thessaloniki, 54124 Thessaloniki, Greece; konnidin@windowslive.com (K.D.);; 3 Department of ENT Surgery, General Hospital of Mytilene ‘Vostaneio’, 81132 Mytilene, Greece; 4 Department of Anesthesiology and Pain Medicine, General Hospital of Drama, 66132 Drama, Greece; 5 Department of General Surgery, Landesspital Liechtenstein, FL-9490 Vaduz, Liechtenstein

**Keywords:** clear cell renal cell carcinoma, thyroid metastasis, renal cell carcinoma, fine-needle aspiration, immunohistochemistry, thyroidectomy

## Abstract

**Background and Clinical Significance**: Thyroid metastasis from clear cell renal cell carcinoma (ccRCC) is uncommon and may occur after radical nephrectomy despite no evidence of residual or metastatic disease at that time. Diagnosis can be challenging because imaging and fine-needle aspiration (FNA) may mimic benign or primary thyroid lesions. **Case Presentation**: A 55-year-old man underwent left radical nephrectomy for WHO/ISUP grade 3 ccRCC with focal grade 4 differentiation. Two years later, surveillance ultrasonography detected a suspicious lesion involving the thyroid isthmus and left lobe. FNA was interpreted as Bethesda II (benign), but metastatic ccRCC was subsequently confirmed after total thyroidectomy. Histopathology showed focal vascular and capsular invasion. Tumor reached the margin of the main thyroid specimen, whereas separately submitted perithyroidal soft tissue had tumor-free margins, resulting in a final R0 resection. The tumor was positive for CK8/18, PAX8, EMA, vimentin, and CD10 and negative for TTF-1, thyroglobulin, calcitonin, and CD5. Postoperative 18F-FDG PET/CT showed no additional metastatic disease. **Conclusions**: Benign thyroid cytology should be interpreted cautiously in patients with previous ccRCC when clinical or imaging findings remain suspicious. Diagnosis requires integration of oncological history, imaging, histopathology, and immunohistochemistry. Surgical resection may provide local disease control in selected patients, with surveillance.

## 1. Introduction and Clinical Significance

Advances in the treatment of renal cell carcinoma together with structured postoperative surveillance have increased the detection of incidental thyroid lesions during long-term follow-up. Renal cell carcinoma (RCC) accounts for approximately 2–3% of all adult malignancies and is characterized by highly variable biological behavior and an unpredictable metastatic pattern [1]. Clear cell renal cell carcinoma (ccRCC), the most common histological subtype, frequently metastasizes to the lungs, bones, liver, lymph nodes, and brain. In contrast, thyroid metastases are distinctly uncommon in clinical practice despite the rich vascularity of the thyroid gland and represent only a small proportion of secondary thyroid malignancies [1,2,3].

Thyroid involvement may occur many years after radical nephrectomy, even when there was no evidence of residual or metastatic disease at the time of surgery, and is often discovered incidentally during routine surveillance or during evaluation of a newly detected thyroid nodule [2,3]. Because both the clinical presentation and ultrasonographic appearance frequently resemble those of primary thyroid lesions, distinguishing metastatic RCC from benign or primary thyroid disease remains challenging. Furthermore, fine-needle aspiration (FNA), although considered the cornerstone of thyroid nodule evaluation, may occasionally yield false-negative or inconclusive results in metastatic RCC, potentially delaying definitive diagnosis [4].

Definitive diagnosis relies on histopathological examination supported by appropriate immunohistochemistry. Surgical resection is an important treatment option for apparently isolated thyroid metastasis when technically feasible [1,2].

This report adds to the limited contemporary literature on isolated thyroid metastasis from high-grade ccRCC by highlighting the diagnostic pitfall of benign Bethesda II cytology, the value of comprehensive immunohistochemical characterization, and the disease-free course observed during the available follow-up after R0 resection.

## 2. Case Presentation

A 55-year-old man with no family history of endocrine or hereditary malignancies underwent left radical nephrectomy in February 2020 for a renal mass detected during diagnostic evaluation. Histopathological examination revealed high-grade clear cell renal cell carcinoma measuring 4.5 × 4.0 × 4.0 cm. The tumor was classified as WHO/ISUP grade 3 with focal grade 4 differentiation. Immunohistochemical analysis demonstrated diffuse positivity for EMA, vimentin, and CD10, whereas CK7 was negative. The Ki-67 proliferation index was approximately 10–15%. The diagnosis of ccRCC was established on the basis of the characteristic histomorphological and immunophenotypic findings. Surgical margins, the ureter, renal pelvis, and major vascular structures were free of tumor involvement. According to the AJCC 8th edition TNM classification [5], the primary renal tumor was staged as pT1b pN0 cM0. Histopathological examination showed no evidence of renal sinus fat, perinephric fat, or major vascular invasion.

**Figure 1:**
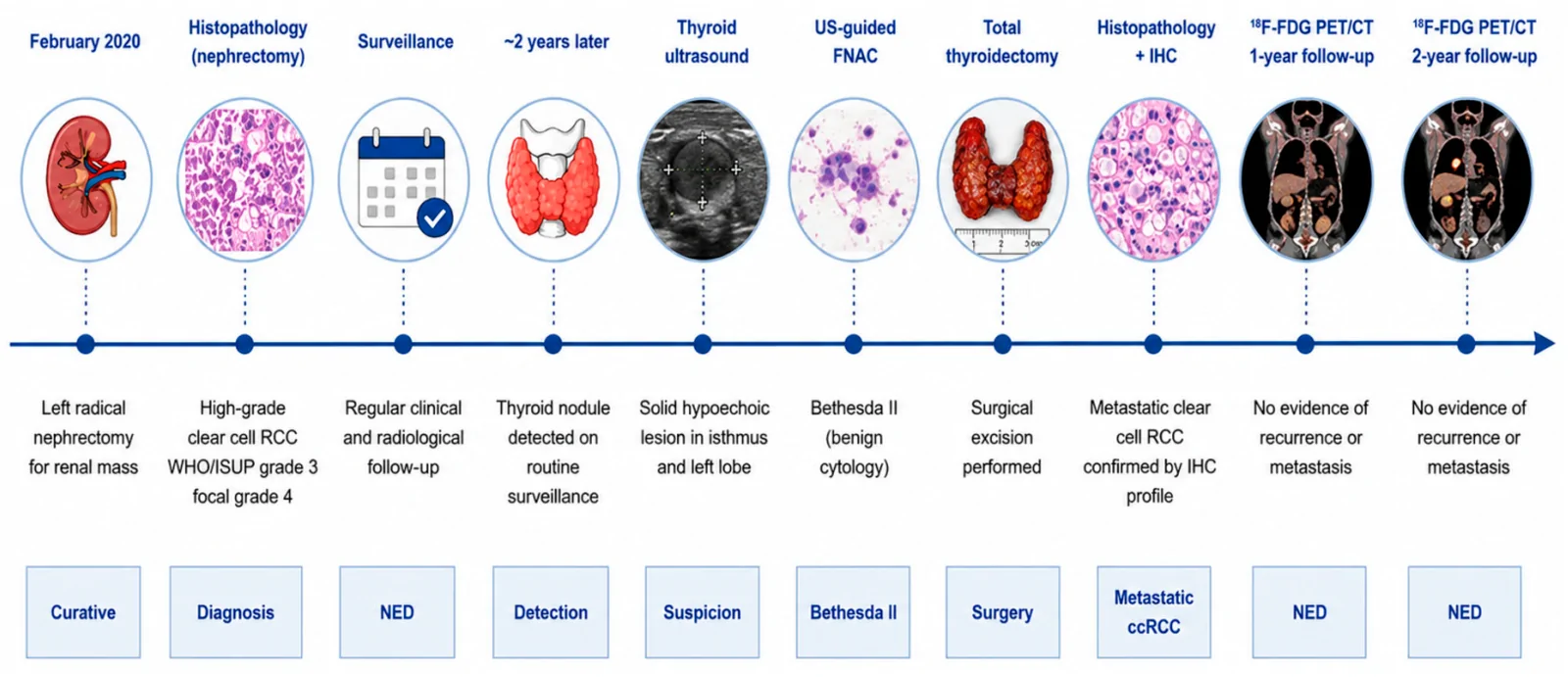
Clinical timeline from radical nephrectomy for high-grade clear cell renal cell carcinoma (ccRCC) to the diagnosis of isolated thyroid metastasis, total thyroidectomy, and disease-free follow-up confirmed by serial 18F-FDG PET/CT.

Following nephrectomy, the patient was enrolled in a structured surveillance program. Serial clinical examinations and cross-sectional imaging showed no evidence of local recurrence or distant metastatic disease. Computed tomography of the chest, abdomen, and pelvis performed during follow-up demonstrated no pulmonary, hepatic, lymph node, or osseous metastases. Abdominal ultrasonography performed subsequently also showed no evidence of recurrent disease, confirming stable postoperative findings (Figure 1).

**Figure 2:**
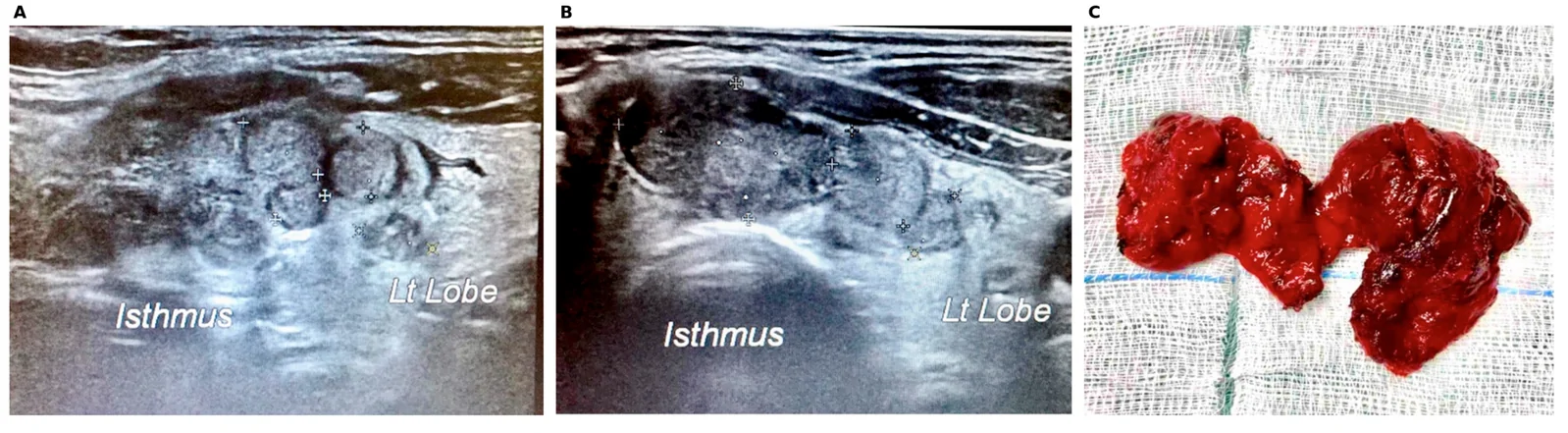
Preoperative ultrasonographic and gross pathological findings. (**A**,**B**) Transverse and longitudinal thyroid ultrasonography demonstrating a heterogeneous hypoechoic solid lesion centered within the thyroid isthmus with extension into the lower pole of the left thyroid lobe. (**C**) Gross thyroidectomy specimen showing the nodular lesion involving the lower pole of the left thyroid lobe.

Approximately two years after nephrectomy, while the patient remained asymptomatic, routine neck ultrasonography incidentally demonstrated a solid hypoechoic lesion involving the thyroid isthmus with extension toward the lower pole of the left thyroid lobe (Figure 2A,B). The lesion showed irregular margins and heterogeneous echogenicity, without evidence of cervical lymphadenopathy. At the time of thyroid evaluation, the patient was clinically euthyroid, thyroid function tests were within normal limits, and there was no known history of pre-existing thyroid disease. Detailed information regarding lesion dimensions, vascularity, calcifications, shape, and a standardized ultrasound risk classification was not available in the clinical documentation for this report. Ultrasound-guided fine-needle aspiration cytology was subsequently performed. Cytological examination revealed abundant colloid with scattered follicular epithelial cells and no cytological evidence of malignancy, corresponding to Bethesda category II (benign cytology). Despite the benign cytological findings, metastatic disease could not be confidently excluded because of the patient’s history of high-grade ccRCC and the suspicious ultrasonographic appearance of the lesion. No repeat FNA, core-needle biopsy, or cell-block immunohistochemistry was performed before surgery, and surgical excision was pursued to obtain a definitive diagnosis. Total thyroidectomy was selected because the lesion involved the thyroid isthmus with extension into the left lobe. Hemithyroidectomy was considered less appropriate because complete excision while preserving the contralateral thyroid was considered less reliable. Furthermore, if metastatic disease had subsequently been confirmed, a completion thyroidectomy might have been required.

**Figure 3:**
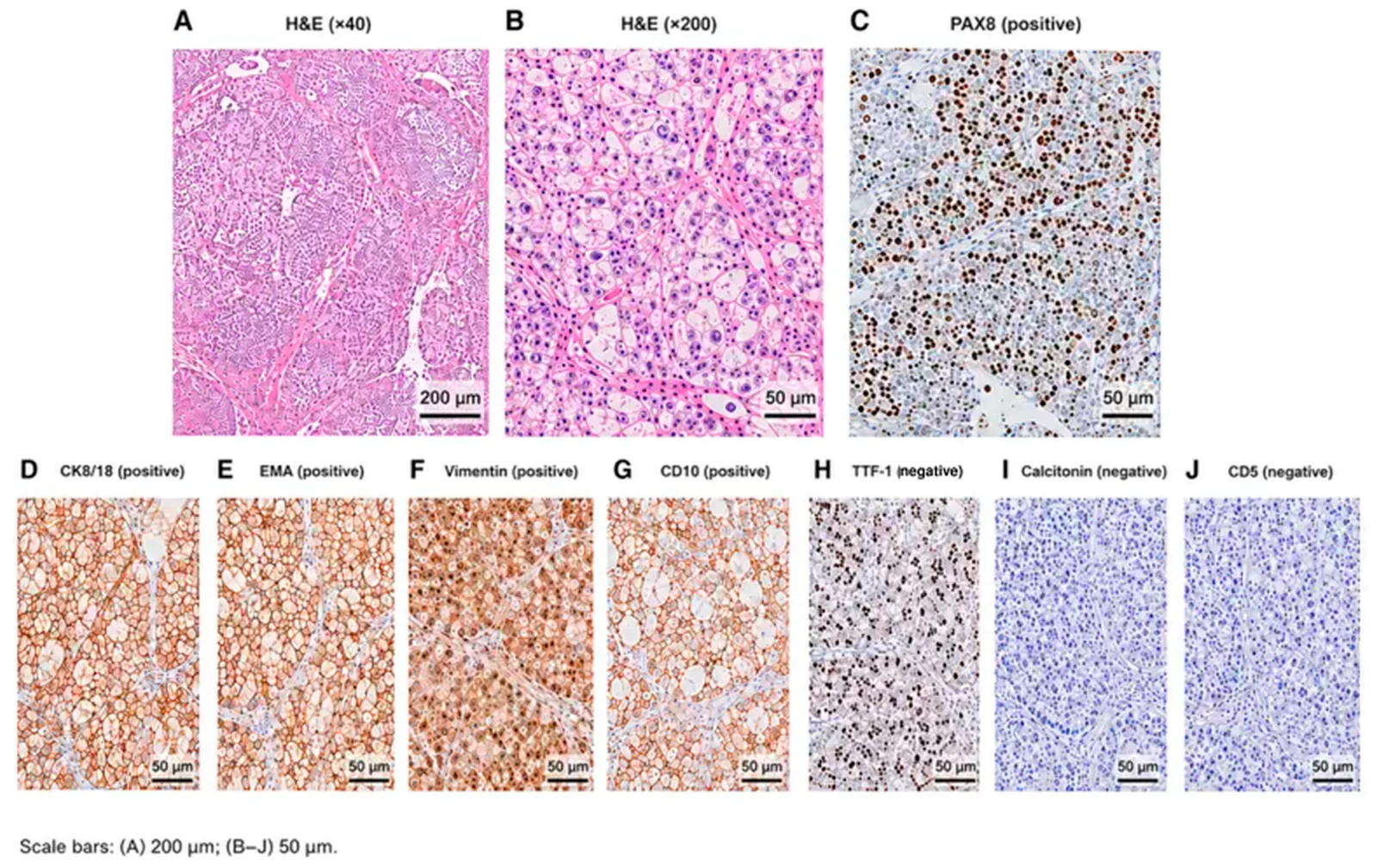
Histopathological and immunohistochemical findings of metastatic clear cell renal cell carcinoma involving the thyroid gland. (**A**) Low-power H&E staining (×40) showing tumor infiltration of the thyroid parenchyma. (**B**) Higher-power H&E staining (×200) demonstrating polygonal clear cells with abundant clear cytoplasm. (**C**–**G**) Immunohistochemical staining showing positivity for PAX8 (**C**), CK8/18 (**D**), EMA (**E**), vimentin (**F**), and CD10 (**G**). (**H**–**J**) Negative staining for TTF-1 (**H**), calcitonin (**I**), and CD5 (**J**). The combined histomorphological and immunophenotypic findings support the diagnosis of metastatic clear cell renal cell carcinoma. Scale bars: 200 μm (**A**) and 50 μm (**B**–**J**).

Gross examination revealed a solid nodular lesion involving the lower pole of the left thyroid lobe (Figure 2C). Microscopic examination demonstrated extensive infiltration of the thyroid parenchyma by metastatic carcinoma composed of nests and alveolar structures of polygonal clear cells with abundant optically clear cytoplasm, distinct cell membranes, and prominent nucleoli (Figure 3A,B). Immunohistochemistry demonstrated positivity for CK8/18, PAX8, EMA, vimentin, and CD10, whereas TTF-1, thyroglobulin, calcitonin, CD5, and synaptophysin were negative, supporting the diagnosis of metastatic ccRCC in conjunction with the characteristic histomorphological findings. CAIX and RCC marker immunostains were not performed. The original nephrectomy specimen was not directly re-reviewed or compared histologically with the thyroid specimen. The diagnosis was based on the characteristic morphology and immunophenotypic profile of the thyroid lesion together with the documented history of ccRCC. Histopathological examination demonstrated multifocal metastatic ccRCC with focal vascular and capsular invasion. Metastatic carcinoma extended to the resection margin of the left thyroid lobe specimen. A separately submitted perithyroidal soft-tissue specimen from the lower pole of the left lobe also demonstrated metastatic ccRCC with identical histomorphological and immunophenotypic features; however, the surgical margins of this additional specimen were free of tumor. Therefore, the final overall margin status was considered microscopically negative (R0). The remaining thyroid parenchyma showed multinodular hyperplasia without additional metastatic foci. No cervical lymph nodes were sampled. No dedicated systemic staging was performed immediately before thyroidectomy because the thyroid lesion had been interpreted as benign on preoperative FNA. Following histopathological confirmation of metastatic ccRCC, whole-body 18F-FDG PET/CT was performed and demonstrated no evidence of additional metastatic disease, supporting the retrospective characterization of the thyroid lesion as an isolated metastasis.

The immediate postoperative course was uneventful. Following histopathological confirmation of metastatic ccRCC, whole-body 18F-FDG PET/CT demonstrated no evidence of additional metastatic disease. One month after thyroidectomy, the patient suffered an acute myocardial infarction. Following multidisciplinary oncological assessment, the recent cardiovascular event, the patient’s overall clinical status, the final R0 resection, and the absence of detectable metastatic disease elsewhere were considered in the decision not to initiate immediate systemic therapy. A structured surveillance strategy was therefore adopted. Clinical assessment was performed every two months, with repeat neck ultrasonography at three months and subsequently every six months until two years after thyroidectomy. Whole-body 18F-FDG PET/CT was performed annually. Throughout follow-up, no evidence of local recurrence or distant metastatic disease was detected, and the patient remained disease-free at the latest evaluation.

## 3. Discussion

Renal cell carcinoma (RCC) is characterized by marked biological heterogeneity and an unpredictable metastatic pattern. Although the lungs, bones, liver, lymph nodes, and brain represent the most common sites of dissemination, the thyroid gland is an uncommon but clinically relevant site of metastasis, particularly from clear cell renal cell carcinoma (ccRCC). Thyroid metastases from ccRCC are predominantly metachronous and may occur months, years, or even decades after nephrectomy [1,2,3]. This prolonged and variable interval reflects the distinctive biological behavior of ccRCC and emphasizes the importance of considering a previous renal malignancy when evaluating a newly detected thyroid lesion. Representative published cases and their clinicopathological characteristics are summarized in Table 1.

The mechanisms underlying thyroid metastasis remain incompletely understood. Hematogenous dissemination, tumor dormancy, and subsequent reactivation have been proposed as potential mechanisms responsible for delayed metastatic recurrence. Despite the rich vascular supply of the thyroid gland, its high oxygen and iodine content and rapid blood flow have traditionally been considered relatively unfavorable for metastatic implantation. Conversely, pre-existing thyroid abnormalities, including multinodular goiter, chronic thyroiditis, and stromal fibrosis, have been suggested to alter the local tissue microenvironment and potentially facilitate metastatic colonization [1,2,3].

Compared with representative cases reported in the literature, the present case is notable for several features. First, thyroid metastasis developed only two years after nephrectomy, whereas many reported thyroid metastases from ccRCC have occurred after substantially longer disease-free intervals, in some cases several years or even decades after treatment of the primary renal tumor [1,2,3]. The present case therefore illustrates that thyroid metastasis should not be regarded exclusively as a late manifestation of ccRCC. Second, although the primary renal tumor was relatively limited anatomically (pT1b), it demonstrated WHO/ISUP grade 3 morphology with focal grade 4 differentiation. This observation does not establish a relative prognostic superiority of histological grade over anatomical stage; rather, it illustrates that metastatic recurrence may occur even after a relatively low-stage primary tumor when adverse histological features are present. Third, the thyroid lesion was initially classified as Bethesda II on FNA despite suspicious ultrasonographic findings, and metastatic disease was established only after surgical excision. Finally, despite the adverse pathological features of the thyroid metastasis, postoperative staging showed no additional metastatic disease, and the patient remained free of detectable recurrence during the available follow-up period. Taken together, these findings illustrate the heterogeneity of thyroid metastases from ccRCC in terms of timing, primary tumor characteristics, diagnostic presentation, and subsequent clinical course.

A further distinctive feature was the discrepancy between the suspicious ultrasonographic appearance and the initially benign cytological interpretation. High-resolution ultrasonography remains the first-line imaging modality for thyroid nodules; however, thyroid metastases from ccRCC lack pathognomonic sonographic characteristics and may resemble either benign thyroid nodules or primary thyroid malignancies. Reported findings commonly include a solid hypoechoic appearance, heterogeneous echogenicity, hypervascularity, and irregular margins [4,6,7]. In the present case, ultrasound-guided fine-needle aspiration (FNA) was interpreted as Bethesda category II, indicating benign cytology. The diagnosis of metastatic ccRCC was established only after surgical excision and histopathological examination. Thus, the initial Bethesda II cytological interpretation was retrospectively recognized as false-negative after histopathological confirmation of metastatic ccRCC.

Although FNA remains the cornerstone of thyroid nodule assessment, its diagnostic performance may be lower in metastatic lesions than in primary thyroid tumors. Sampling error, hemorrhagic aspirates, marked vascularity, limited tumor cellularity, and cytological overlap with primary thyroid lesions may contribute to nondiagnostic or false-negative results [4,6,7]. The present case therefore reinforces an important diagnostic principle: benign thyroid cytology should not be interpreted in isolation when there is persistent clinical or radiological suspicion, particularly in a patient with a previous history of ccRCC. Instead, cytological findings should be integrated with the patient’s oncological history, imaging characteristics, and overall clinical context.

Definitive diagnosis in the present case relied on the integration of histomorphological and immunohistochemical findings. The metastatic tumor demonstrated positivity for CK8/18, PAX8, EMA, vimentin, and CD10, whereas TTF-1, thyroglobulin, calcitonin, CD5, and synaptophysin were negative. The absence of TTF-1 and thyroglobulin expression argued against a primary thyroid follicular-derived neoplasm, while the characteristic clear-cell morphology and renal immunophenotype supported metastatic ccRCC [3,10,11]. PAX8, although positive, is expressed in both renal epithelial and thyroid follicular-derived tumors and therefore cannot independently establish the site of origin. Although CAIX and RCC marker may provide additional support for renal origin, these immunostains were not performed in the present case. Accordingly, the diagnosis was established by integrating the characteristic clear-cell morphology, the available immunophenotypic profile, the absence of TTF-1 and thyroglobulin expression, and the patient’s previous oncological history rather than relying on any single marker.

These diagnostic features distinguish the present case from a routine evaluation of a thyroid nodule and constitute its principal educational value. The relatively short interval between nephrectomy and thyroid metastasis, the occurrence of metastatic disease after a relatively low-stage but high-grade primary tumor, the benign Bethesda II cytological interpretation, and the discrepancy between cytology and the subsequent histopathological diagnosis collectively demonstrate important diagnostic pitfalls. In this setting, awareness of the patient’s oncological history and careful clinicopathological correlation were essential to reaching the correct diagnosis.

Published evidence supports surgical resection as an important therapeutic option for appropriately selected patients with apparently isolated thyroid metastasis from RCC when technically feasible [1,2,12,13,14,15]. In addition to providing a definitive histopathological diagnosis, retrospective series suggest that surgery may provide effective local disease control, particularly in patients without widespread metastatic disease [12,13,14,15]. These data are derived primarily from retrospective series and selected patient populations and should therefore be interpreted cautiously. Neither the American Thyroid Association nor the European Association of Urology provides disease-specific recommendations for thyroid metastases from RCC; management should therefore be individualized according to the anatomical extent of disease, overall metastatic burden, patient fitness, and feasibility of local treatment [12,13,14,15,16].

Systemic therapies, including immune checkpoint inhibitors and tyrosine kinase inhibitors, have an established role in advanced or disseminated metastatic RCC [12,16]. In patients with apparently isolated thyroid involvement, postoperative management should be individualized according to systemic disease burden, pathological findings, surgical outcome, comorbidities, and overall clinical status. In the present case, although metastatic carcinoma extended to the resection margin of the main left thyroid lobe specimen, the separately submitted perithyroidal soft-tissue specimen had tumor-free margins, resulting in a final overall microscopically negative (R0) resection. Postoperative 18F-FDG PET/CT demonstrated no evidence of additional metastatic disease. One month after thyroidectomy, the patient suffered an acute myocardial infarction. Following multidisciplinary oncological assessment, the recent cardiovascular event and the patient’s overall clinical status were considered important factors against immediate systemic treatment. In view of the final R0 resection, the absence of detectable metastatic disease elsewhere, and the patient’s comorbid condition, structured surveillance was selected. Serial postoperative imaging subsequently demonstrated no evidence of recurrent or distant metastatic disease during the available follow-up period.

Long-term surveillance remains particularly important in ccRCC because metastatic recurrence may occur after prolonged disease-free intervals. Current European Association of Urology guidelines recommend risk-adapted postoperative surveillance following treatment of RCC, with follow-up intensity determined by the individual patient’s recurrence risk [16]. In the present case, serial clinical assessment, neck ultrasonography, and whole-body 18F-FDG PET/CT examinations demonstrated no evidence of recurrent or additional metastatic disease during follow-up. Nevertheless, the approximately two-year follow-up after thyroidectomy remains relatively limited in the context of the potentially prolonged natural history of ccRCC, and continued surveillance is warranted.

As a single-case report, the present observation cannot establish prognostic relationships or determine the relative importance of tumor stage, histological grade, cytological findings, or specific therapeutic approaches. Its value lies instead in highlighting a clinically relevant diagnostic pitfall and demonstrating the importance of maintaining suspicion for metastatic ccRCC in patients with a previous renal malignancy who develop a new thyroid lesion, even when initial cytology is benign. Direct comparison with previously published cases further illustrates the considerable heterogeneity in the timing, presentation, diagnostic findings, and clinical course of thyroid metastases from ccRCC.

## 4. Conclusions

Thyroid metastasis should be included in the differential diagnosis of newly detected thyroid nodules in patients with a history of clear cell renal cell carcinoma, irrespective of the interval since nephrectomy. Although fine-needle aspiration remains the cornerstone of thyroid nodule evaluation, false-negative cytological results may occur and should not override suspicious clinical or radiological findings. Definitive diagnosis requires integration of the patient’s oncological history, imaging findings, histomorphology, and immunohistochemical profile. In appropriately selected patients with apparently isolated thyroid metastasis, surgical resection may provide effective local disease control, as suggested by the available literature. Treatment decisions should be individualized according to disease extent, surgical findings, comorbidities, and overall clinical status.

## Figures and Tables

**Table 1:** Clinicopathological comparison of representative published cases of thyroid metastasis from renal cell carcinoma and the present case.

Study/Case	Age/Sex	Interval After Nephrectomy	Thyroid Presentation	Preoperative Cytology	Surgical Management	Diagnostic IHC Profile	Follow-Up After Thyroidectomy	Outcome
Jia et al., 2023 [6] Case 1	78/F	12 years	Hoarseness, dyspnea; bilateral thyroid nodules with cervical lymphadenopathy	FNA and CNB nondiagnostic	Total thyroidectomy	CD10 positive (reported)	NR	NR
Jia et al., 2023 [6] Case 2	54/M	8 years	Goiter with tracheal compression; partially cystic left thyroid nodule	NR	Total thyroidectomy	NR	NR	NR
Jia et al., 2023 [6] Case 3	65/M	7 years	Goiter with tracheal compression; partially cystic thyroid nodule	NR	Total thyroidectomy	NR	NR	NR
Xu et al., 2024 [7] Case 1	59/M	9 years	Multiple thyroid nodules; cervical and pulmonary nodules	Initially interpreted as follicular carcinoma	Total thyroidectomy and right neck dissection	CAIX+, CD10+, PAX8+, Tg−, TTF-1−	Latest follow-up	No progression
Xu et al., 2024 [7] Case 2	62/F	NR	Incidental anterior neck mass; bilateral thyroid nodules	NR	Right lobectomy and left lumpectomy	CD10+, PAX8+, pan-CK+, TTF-1−	NR	NR
Hryshchyshyn et al., 2024 [8]	58/F	13 years	Two thyroid nodules (3.5 and 1.5 cm); TI-RADS 3	Bethesda IV	Total thyroidectomy	NR	Reported	Local and distant recurrence
Khalafi-Nezhad et al., 2024 [9]	75/F	13 years	Late-onset thyroid nodules	NR	NR	Metastatic RCC confirmed	NR	NR
Present case	55/M	About 2 years	Incidental solid hypoechoic lesion involving the thyroid isthmus with extension into the left lobe	Bethesda II (benign); retrospectively recognized as false-negative after histopathological diagnosis	Total thyroidectomy	CK8/18+, PAX8+, EMA+, vimentin+, CD10+; TTF-1−, Tg−, calcitonin−, CD5−, synaptophysin−	About 2 years; clinical review, neck ultrasonography, and annual PET/CT	No clinically or radiologically detected recurrence at approximately 2 years; final overall margin status R0

Abbreviations: CAIX, carbonic anhydrase IX; CNB, core-needle biopsy; FNA, fine-needle aspiration; IHC, immunohistochemistry; NR, not reported; pan-CK, pancytokeratin; PET/CT, positron emission tomography/computed tomography; RCC, renal cell carcinoma; Tg, thyroglobulin.

## Data Availability

The original contributions presented in this study are included in the article. Further inquiries can be directed to the corresponding author.

## Abbreviations

The following abbreviations are used in this manuscript:

CASTLE	Carcinoma Showing Thymus-Like Elements
ccRCC	Clear cell renal cell carcinoma
CT	Computed tomography
EAU	European Association of Urology
EMA	Epithelial membrane antigen
FNA	Fine-needle aspiration
H&E	Hematoxylin and eosin
PET/CT	Positron emission tomography/computed tomography
PAX8	Paired box gene 8
RCC	Renal cell carcinoma
WHO/ISUP	World Health Organization/International Society of Urological Pathology
